# Prevalence, Causes and Socio-Economic Determinants of Vision Loss in Cape Town, South Africa

**DOI:** 10.1371/journal.pone.0030718

**Published:** 2012-02-20

**Authors:** Nicky Cockburn, David Steven, Karin Lecuona, Francois Joubert, Graeme Rogers, Colin Cook, Sarah Polack

**Affiliations:** 1 Department of Ophthalmology, Groote Schuur Hospital, Cape Town, South Africa; 2 Eerste River Hospital, Cape Town, South Africa; 3 London School of Hygiene and Tropical Medicine, London, United Kingdom; University of Buea, Cameroon

## Abstract

**Purpose:**

To estimate the prevalence and causes of blindness and visual impairment in Cape Town, South Africa and to explore socio-economic and demographic predictors of vision loss in this setting.

**Methods:**

A cross sectional population-based survey was conducted in Cape Town. Eighty-two clusters were selected using probability proportionate to size sampling. Within each cluster 35 or 40 people aged 50 years and above were selected using compact segment sampling. Visual acuity of participants was assessed and eyes with a visual acuity less than 6/18 were examined by an ophthalmologist to determine the cause of vision loss. Demographic data (age, gender and education) were collected and a socio-economic status (SES) index was created using principal components analysis.

**Results:**

Out of 3100 eligible people, 2750 (89%) were examined. The sample prevalence of bilateral blindness (presenting visual acuity <3/60) was 1.4% (95% CI 0.9–1.8). Posterior segment diseases accounted for 65% of blindness and cataract was responsible for 27%. The prevalence of vision loss was highest among people over 80 years (odds ratio (OR) 6.9 95% CI 4.6–10.6), those in the poorest SES group (OR 3.9 95% CI 2.2–6.7) and people with no formal education (OR 5.4 95% CI 1.7–16.6). Cataract surgical coverage was 68% in the poorest SES tertile (68%) compared to 93% in the medium and 100% in the highest tertile.

**Conclusions:**

The prevalence of blindness among people ≥50 years in Cape Town was lower than expected and the contribution of posterior segment diseases higher than previously reported in South Africa and Sub Saharan Africa. There were clear socio-economic disparities in prevalence of vision loss and cataract surgical coverage in this setting which need to be addressed in blindness prevention programs.

## Introduction

Globally there are an estimated 45 million people who are blind, with the highest of prevalence of blindness found in Africa [1], [2]. VISION 2020 is a joint initiative between the World Health Organisation (WHO) and the International Agency for the Prevention of blindness which aims to eliminate avoidable blindness by the year 2020 [3]. WHO estimates from 2002 indicate the prevalence of blindness in Sub-Saharan Africa (SSA) is 1% in all ages and 9% among people ≥50 years [1]. However, recent population based surveys suggest that the prevalence of blindness in the region may be considerably lower [4], [5], [6], [7], [8], [9].

The majority of blindness surveys in SSA are from rural settings [4], [5], [6], [7], [9]. Data are lacking from urban, middle income settings such as Cape Town in South Africa. Studies in rural South Africa conducted more than 20 years ago estimated the prevalence of blindness to be between 0.6% and 1.0% [10], [11] for all ages and 1.4–3.2% among people aged ≥40 years [12], [13] with cataract accounting for 55–59% of blindness [10], [11]. However, extrapolating findings from these and other surveys in SSA to Cape Town may not be appropriate given the likely higher proportion of older persons [14], higher prevalence of diabetes [15] and greater availability of ophthalmic services in this urban setting; all factors which influence the epidemiology of vision loss. Reliable, up-to-date data on the prevalence and cause of blindness is needed in Cape Town order to appropriately plan Vision 2020 blindness prevention programmes.

Although vision loss is widely acknowledged to have important demographic and socio-economic determinants, there is relatively limited quantitative research exploring the nature of the association in different settings [16]. Poverty and blindness are thought to be cyclically linked, with poverty increasing the risk of becoming blind [17] and blindness exacerbating poverty through limiting opportunities to engage in income generating activities [18], [19] At country level, the prevalence of blindness is higher in poor countries compared to in wealthier countries [1] and within countries, limited data suggest that the poor are more likely to be blind [20], [21]. Studies in South Africa have shown that socioeconomic status (SES) is associated with access to [22], [23] and satisfaction with [24] health care. However, the role of these factors in vision loss in South Africa is not known. Understanding this association and identifying at risk groups can help to inform the equitable and efficient use of limited eye care resources.

The aim of this study was to estimate the prevalence and causes of blindness and visual impairment in Cape Town and to investigate the relationship between socio economic factors and vision loss.

## Methods

### Study setting

This survey was undertaken in Cape Town between September and November 2010. Cape Town has a population of approximately 3.7 million [25] 76% of whom have no medical insurance and are dependent on the public sector [26]. There are three public sector tertiary level hospitals (two for adults, 1 for children) and one district level hospital providing eye care in Cape Town. There are 12 ophthalmologists (excluding residents/registrars) working full time in the public sector and considerably more (54) working in the private sector.

### Sample-size

The prevalence of blindness in people aged >50 years was estimated to be 3% [4],[5],[6],[7],[9]. Allowing a worst acceptable result of 1%, a design effect of 1.5, confidence of 95%, and a 10% non-response rate, a minimum sample size of 3077 was required. In total 77 clusters of 40 people were required for this survey. For logistical reasons we selected 36 clusters of 35 people and 46 clusters of 40 people (total 82 clusters).

### Sampling procedure

We used the data from the 2001 census as the sampling frame, updated with expected population growth estimates [25]. Clusters (‘small areas’) were selected using probability proportionate to size sampling. Two of the selected clusters were considered unsafe for the survey team to visit and were replaced by selecting the next small area listed on the sampling frame. Within clusters, households were selected using compact segment sampling [27], [28]. Aerial maps displaying houses, street names and boundaries were obtained for all selected clusters. Based on the housing density, clusters were divided into segments each including approximately 35 or 40 (depending on cluster size) people aged ≥50 years. One of the segments was selected at random by drawing lots, and all eligible people were included sequentially until the required cluster size was achieved. If the target number was not reached then another segment was chosen at random and sampling continued. The survey team visited households door-to-door to identify eligible people. Absentees were re-visited on the same day in the evening. If more than 10% of the cluster were absent after the second visit, the households were re-visited the following Saturday.

### Data collected and protocol for ocular examination

A standardised survey form was completed for each eligible person which included data on examination status, presenting and pinhole vision, lens examination, principal cause of visual impairment, history of visual impairment from a relative or neighbour if subject was not available, why cataract operation had not been carried out (in people with VA<6/60 from cataract in either eye) and details about the operation in those who had had cataract surgery.

Visual acuity (VA) was measured with available spectacle correction by a doctor using a tumbling “E” chart, with optotype size 6/18 (20/60) on one side and size 6/60 (20/200) on the other side, at a distance of 6 meters. If VA was <6/18 in either eye pinhole vision was also measured. Blindness was defined as VA<3/60, severe visual impairment (SVI) as VA<6/60–3/60 and visual impairment as VA<6/18–6/60.

The lens status of all participants was assessed by an ophthalmologist using a direct ophthalmoscope in a dark environment. People with pinhole corrected VA<6/18 in either eye underwent further examination by the ophthalmologist using a direct ophthalmoscope to ascertain the cause of vision loss. According to WHO convention the major cause was assigned to the primary disorder or, if there were two existing primary disorders, to the one that is easiest to treat [29]. The pupil was dilated to allow adequate visualisation of the posterior pole if the cause of visual impairment could not be determined by examination with an undilated pupil.

### Socioeconomic and demographic variables

Data were collected on age, gender and highest level of education completed. We used a household SES index as a proxy for wealth. Participants were asked about household ownership of 14 durable items [30] and an SES index was created from these variables using principal component analysis [31]. The index was divided into tertiles of wealth from poorest (lowest SES index) to wealthiest (highest SES).

### Training

Five teams, each consisting of one ophthalmologist and one or two Community Based Eye Workers received 5 days training. The inter-observer agreement for VA measurement, lens examination and cause of vision loss was assessed and the kappa value was of an acceptable standard (>0.6) for each team.

### Data analysis

All data were double entered. Data on prevalence and causes of vision loss and details of cataract surgery were analysed using the automated RAAB data package. Confidence intervals are calculated taking into account the cluster sampling design. Cataract surgical coverage (CSC) was defined as the proportion of people needing cataract surgery who had received it.

For persons this is calculated as:
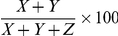
In which:

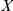
 = Number of people with unilateral (pseudo)aphakia and visual impairment from cataract in the other eye

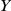
 = Number of people with bilateral (pseudo)aphakia

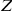
 = Number of people with bilateral visual impairment from cataract

VA before surgery is unknown and therefore these calculations were undertaken assuming, in turn, that only patients with VA<3/60, <6/60 and <6/18 undergo cataract surgery. CSC was also calculated for eyes.

Stata 11 software was used to analyse the association between vision loss (VA<6/18) demographic (age and gender) and socio-economic variables (SES, and education). We used vision loss rather than blindness as the dependent variable because of the small number of people who were blind. Associations between age, gender, SES and education were assessed through logistic regression analysis using the svy command in STATA to account for the cluster sampling design. To explore the independent association of SES and education with vision loss a multivariate logistic regression analysis was undertaken including all the variables in the model. We also calculated the cataract surgical rate stratified by the different SES groups.

### Ethical considerations

Informed written consent was obtained from all participants. Ethical approval for this work was granted by University of Cape Town Health Sciences Human Ethics Committee (Cape Town, South Africa) and London School of Hygiene and Tropical Medicine (London, UK). All people with ophthalmic conditions requiring treatment or further investigation were referred.

## Results

### Prevalence and causes of vision loss

Out of 3100 eligible people, 2750 (89%) were examined, 169 (5.5%) were not available, 170 (5.5%) refused and 11 (0.4%) were unable to communicate. The mean age of people who were examined and refused were similar (62.7 years, 95% CI 62.4–63.1 and 63.4 years, 95% CI 61.9–64.8 respectively), while those who were not available were slightly younger (59.1 years, 95% CI 57.8–60.4) and those unable to communicate were older (73.5 years 95% CI 66.8–80.1). The age distribution of the sample was similar to that of the census (table 1), however men were slightly under-represented compared to census data.

**Table 1:** Age and gender distribution of the survey sample and census population.

	Male				Female				Total			
Age	Sample		Census		Sample		Census		Sample		Census	
50–59	464	(45%)	134,296	(52%)	773	(45%)	134138	(44%)	1,237	(45%)	268,434	(48%)
60–69	322	(31%)	78,675	(31%)	494	(29%)	99,909	(33%)	816	(30%)	178,584	(32%)
70–79	178	(17%)	31,948	(13%)	290	(17%)	55,182	(18%)	468	(17%)	87,130	(16%)
80+	68	(7%)	11,336	(4%)	155	(9%)	15,944	(5%)	223	(8%)	27,280	(5%)

The prevalence of bilateral blindness (presenting vision) in the survey sample was 1.4% (95% CI 0.9–1.8), severe visual impairment (SVI) was 0.9% (0.6–1.4) and visual impairment (VI) was 4.9% (4.1–5.7) (table 2).

**Table 2:** Estimated prevalence of blindness (VA<3/60), severe visual impairment (VA<6/60–3/60) and visual impairment (VA<6/80–6/60) in Cape Town by person and eyes with available correction.

	Male		Female		Total	
VA with available correction	N	Prevalence (95% CI)	n	Prevalence (95% CI)	n	Prevalence (95% CI)
Blind						
Persons	11	1.1% (0.4–1.7)	26	1.5% (0.9–2.1)	37	1.4% (0.9–1.8)
Eyes	94	4.5% (3.5–5.5)	150	4.4% (3.4–5.3)	242	4.4% (3.9–4.9)
Severe visual impairment						
Persons	6	0.6% (0.1–1.0)	21	1.2% (0.7–1.7)	27	0.9% (0.6–1.4)
Eyes	24	1.2% (0.7–1.7)	72	2.1% (1.5–2.7)	96	1.8% (1.4–2.1)
Visual impairment						
Persons	43	4.1% (2.9–5.2)	91	5.3% (4.2–6.4)	134	4.9% (4.1–5.7)
Eyes	122	5.9% (4.8–7.0)	248	7.2% (6.1–8.4)	372	6.8% (6.1–7.4)

CI, Confidence Interval; VA, Visual Acuity;

NB: data for persons refers to VA in the better eye.

Posterior segment diseases were the leading cause of blindness (65%) and were responsible for just over a third of SVI (37%) and one fifth of VI (Table 3). Diabetic retinopathy accounted for 8%, 11% and 2% of blindness, SVI and VI respectively and glaucoma for 11%, 7% and 6%. The remaining posterior segment diseases included age related macular degeneration (ARMD), optic atrophy, trauma and macular hole. Cataract was the second leading cause of blindness (27%) and the leading single cause of SVI (37%). Refractive errors were responsible for 22% of SVI and were the leading cause of VI (50%). Phthisis, cataract surgical complications and other corneal scar were rare as causes of vision loss (<4%). Avoidable causes, which include causes that are treatable or preventable (i.e. operated and un-operated cataract, pthysis, refractive error and corneal scar), made up the 35% of all blindness, 63% of SVI and 79% of VI. If adequate diagnostic and treatment services for DR and glaucoma are in place, so that visual impairment from these conditions is also preventable, avoidable causes rise to 54% of blindness, 81% of SVI and 87% of VI.

**Table 3:** Principal cause of bilateral blindness (VA<3/60), bilateral severe visual impairment (VA<6/60–3/60) and bilateral visual impairment (VA<6/80–6/60) among persons with available correction.

	Blindness		Severe Visual impairment		Visual impairment	
	n	%	n	%	n	%
Refractive error	0	(0%)	6	(22%)	67	(50%)
Cataract, untreated	10	(27%)	10	(37%)	36	(27%)
Aphakia uncorrected	1	(3%)	0	(0%)	0	(0%)
Cataract surgical complications	0	(0%)	0	(0%)	2	(2%)
Phthisis	1	(3%)	0	(0%)	0	(0%)
Other corneal scar	1	(3%)	1	(3%)	1	(1%)
**Avoidable vision loss** a	**13**	**(35%)**	**17**	(**63%**)	**106**	(**80%**)
Diabetic retinopathy	3	(8%)	3	(11%)	3	(2%)
Glaucoma	4	(11%)	2	(7%)	8	(6%)
Age related macular degeneration	4	(11%)	2	(7%)	2	(2%)
Other posterior segment	13	(35%)	7	(13%)	24	(18%)
**Total posterior segment**	**24**	(65%)	**11**	(**37%**)	**27**	(**20%**)

VA, Visual Acuity.

a Avoidable causes: Cataract (including un-operated cataract and postoperative complications), refractive error, trachoma, and other causes of corneal scars.

Extrapolating the data to the age and gender distribution of the population using the census data, it is estimated that are about 6500 people with bilateral blindness in Cape Town, 5000 with SVI and 24,000 with VI. Of these there are an estimated 1150 people with bilaterally blinding cataract and 14,800 eyes blind from cataract, 1100 people and 3600 eyes with SVI from cataract and 5,600 people and 14,700 eyes VI from cataract. The age and gender adjusted prevalence was 1.2% for blindness, 1.0% for SVI and 4.2% of VI.

Cataract surgical coverage was high for people and eyes, with 96% of people who needed surgery at VA<3/60 having received it, 93% at VA<6/60 and 80% at VA<6/18 (Table 4). Coverage was similar for males and females (Table 4). The majority of surgeries (63%) were undertaken at a private hospital and 35% were at a government hospital. Place of surgery varied by level of socio-economic status: 65% of people in the poorest tertile had surgery at a government facility and 35% at a private facility, while among those in the richest tertile 26% attended a government facility and 74% a private facility (data not shown). Nearly all (98%) cataract surgeries were with intra-ocular lenses (IOLs). Out of all operated eyes, 80% had a good outcome (VA>6/18) with available correction, while 12% had borderline outcome (VA<6/18–6/60) and for 9% outcome was poor (VA<6/60, Table 5). With pinhole correction this increased to 84% good outcome, 8% borderline and 8% poor. Of the 72 eyes with poor/borderline outcome, the leading causes of poor or borderline outcome were concomitant eye disease (67%) and lack of spectacle correction (24%).

**Table 4:** Cataract surgical coverage* by persons and eyes.

	Persons	Eyes
**Blind (VA<3/60)**		
Male	96%	82%
Female	96%	83%
Total	96%	83%
**Severe Visual Impairment (VA<6/60)**		
Male	94%	79%
Female	93%	79%
Total	93%	79%
**Visual Impairment (VA<6/18)**		
Male	81%	68%
Female	80%	64%
Total	80%	65%

VA, Visual Acuity.

*Cataract surgical coverage is the proportion of persons (or eyes) with ‘operable’ cataract who have received cataract surgery.

**Table 5:** Visual acuity outcome after cataract surgery (eyes) with available correction.

	Non-IOL eyes		IOL eyes		Total	
Outcome	n	%	n	%	n	%
**Available Correction**						
Good^a^	1	17%	271	80%	272	80%
Borderline^a^	2	33%	39	12%	41	11%
Poor^a^	3	50%	28	8%	31	9%
**Best correction**						
Good^a^	2	33%	287	85%	289	84%
Borderline^a^	1	17%	25	7%	26	8%
Poor^a^	3	50%	26	8%	29	8%

a WHO classification of visual outcome after cataract surgery. Good: VA>6/18; borderline: VA 6/18–6/60; poor: VA<6/60.

For people with cataract causing VA<6/60 in either eye, we asked the reason why they had not attended for surgery. Among the 18 people with bilateral VA<6/60 who reported barriers, responses were evenly distributed (25% each) between being unaware treatment was possible, unaware how to get surgery, no need for surgery felt and fear.

### Demographic and socioeconomic factors and vision loss

There was a positive association between risk of vision loss and increasing age, although this trend was driven by the significantly increased risk among people aged >80 compared to people aged 50–59 years (Odds Ratio (OR) 5.1, 95% CI 3.5–7.6, Table 6). Being female was also associated with increased risk of vision loss (OR 1.4, 95% CI 1.1–1.9). People in the poorest (OR 4.5 95% CI 1.3–3.9) and medium (OR 3.0 95% CI 1.3–3.9) SES tertiles were much more likely to have vision loss compared to those in the wealthiest tertile (p-for-trend<0.001). There was an inverse association between prevalence of vision loss and increasing level of education (p-for-trend<0.001). The association between vision loss and age, SES and education remained significant with multivariate adjustment.

**Table 6:** The relationship between demographic and socio-economic characteristics and vision loss (bilateral VA<6/18 with available correction).

		VA<6/18		Unadjusted	Multivariate adjusted
	Total	n	%	OR (95% CI)	OR (95% CI)^a^
**Age (years)**					
50–59	1240	71	5.7%	Reference	Reference
60–69	817	40	4.9%	0.8 (0.6–1.3)	0.9 (0.6–1.4)
70–79	468	33	7.1%	1.3 (0.8–1.9)	1.3 (0.8–2.1)
80+	223	53	23.8%	5.1 (3.5–7.6)	6.9 (4.6–10.6)
*p-for trend*				<0.001	<0.001
**Gender**					
Male	1034	59	5.7%	Reference	Reference
Female	1712	138	8.1%	1.4 (1.1–1.9)	1.2 (0.9–1.6)
**SES** b **tertile**					
Poorest	890	107	12.0%	4.5 (1.3–3.9)	3.9 (2.2–6.7)
Medium	984	58	6.5%	3.0 (1.3–3.9)	1.8 (1.0–3.3)
Wealthiest	852	25	2.9%	Reference	Reference
*p-for trend*				<0.001	<0.001
**Education**					
None	172	26	15.1%	11.8 (4.0–34.4)	5.4 (1.7–16.6)
Primary	1473	116	7.9%	5.6 (2.2–14.4)	3.3 (1.3–8.6)
Secondary	42	42	5.6%	3.9 (1.5–10.3)	2.9 (1.2–7.4)
Higher	268	4	1.5%	Reference	Reference
*p-for trend*				<0.001	0.008

VA, Visual Acuity.

a Odds ratios from multivariate logistic regression analysis including all listed variables.

b Socio-economic status.

**Figure 1:**
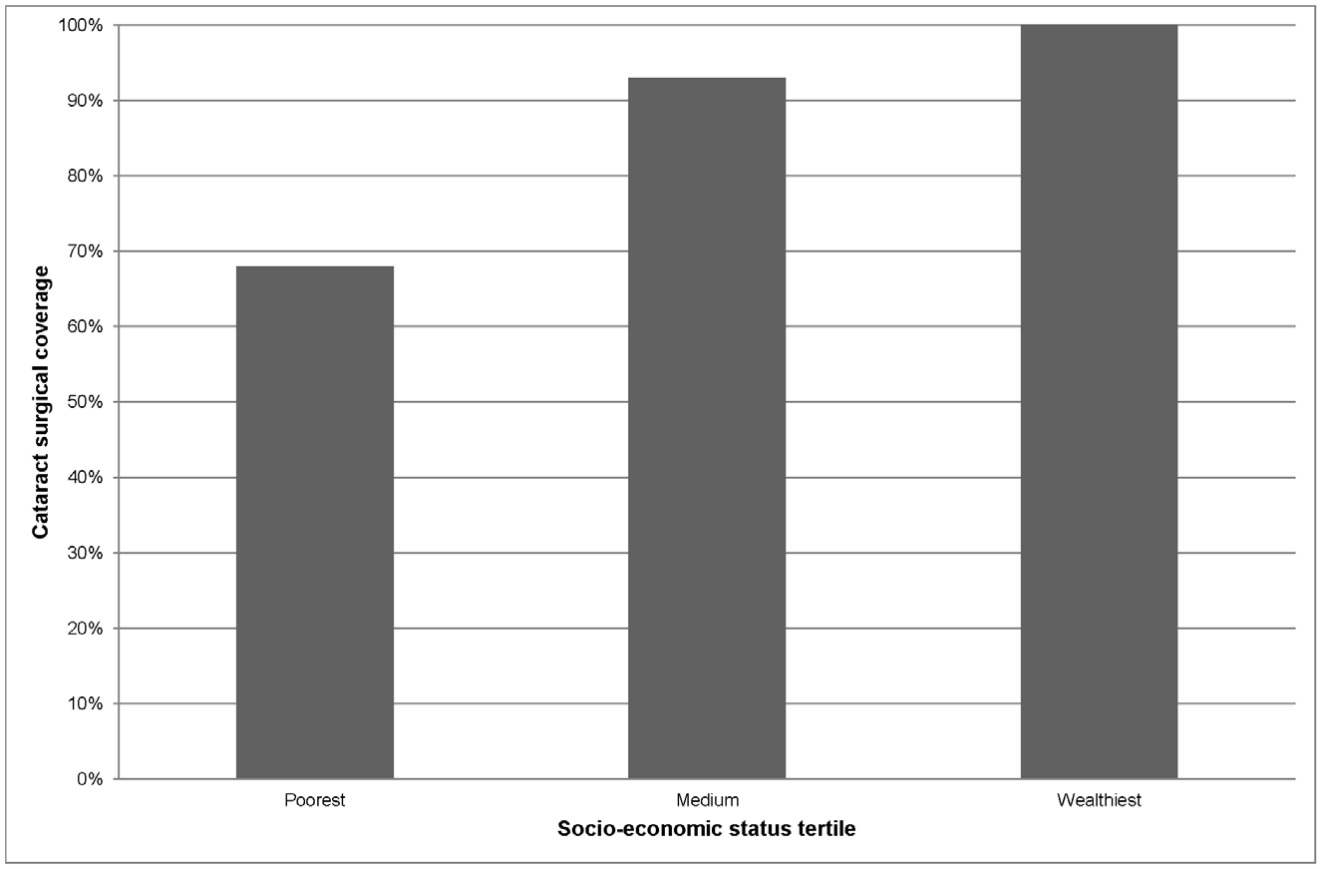
Cataract surgical coverage by socio-economic status. Cataract surgical coverage was defined as the proportion of people needing cataract surgery at VA<6/60 who had received it.

Cataract surgical coverage also varied by SES level. Assuming people have cataract surgery at bilateral VA<6/60, coverage was 100% for those in the wealthiest tertile, 93% for the medium tertile and 68% for the poorest SES tertile (figure 1).

## Discussion

The prevalence of blindness (1.4%), severe visual impairment (0.9%) and visual impairment (4.9%) in people aged ≥50 years Cape Town was considerably lower than the 2002 WHO estimates, a finding which is consistent with other recent surveys conducted in the region [4], [5], [6], [7], [8], [9]. This difference may be due in part to improvements in blindness prevention activities (e.g. increases in cataract surgery coverage) but may also reflect the limited data that were available when the WHO estimates were derived. The prevalence estimates in Cape Town were similar to that in urban Cameroon [8] but lower than that in rural areas in SSA [4], [5], [6], [7], [9]. A lower prevalence in urban settings may be expected as ophthalmic services are likely to be more available and accessible [32]. Surprisingly, the blindness prevalence was similar to that of a survey in rural Northern Transvaal (current day Limpopo Province) conducted more than 20 years ago [10]. This may be due to methodological differences: the authors of the survey in ‘Northern Transvaal’ acknowledge that the estimates should be considered ‘minimum estimates’ because they might have missed cases of blindness [10]. It may also reflect an older population in Cape Town and a higher contribution of posterior segment diseases, which are more difficult to treat.

Posterior segment diseases were responsible for nearly two-thirds of blindness. This is considerably higher than other surveys in the region [4], [5], [6], [7], [8], [9] and is likely to be due in part to the high CSC and consequent low prevalence of cataract blindness in this setting. It may also reflect an older population and possibly higher burden of diabetes and consequent DR. The finding that posterior segment disease is an important cause of vision loss has implications for planning of eye services in Cape Town. Management of DR, glaucoma and ARMD require significantly more resources than cataract, presenting a significant challenge to blindness prevention programs. Treatment for DR and glaucoma is effective, but requires early detection (community based screening), lifelong monitoring and a high level of adherence with therapy to prevent visual loss. For age related macular degeneration there is the added challenge of minimal (in the case of atrophic disease) or limited (for exudative disease) effective treatment. The urgency of this challenge is likely to increase in this setting and throughout Africa as cataract and other avoidable causes of blindness become more under control.

Cataract accounted for 27% of bilateral blindness which is lower than that previously found in South Africa (50–54% in >40 years group [12] [13]) and elsewhere in SSA (42–65% in >50 year group) [4], [5], [6], [7], [9] but similar to that in urban Cameroon [8]. This corresponds with the higher observed CSC in Cape Town. For example, the CSC (for persons <3/60) was found to be 96% in this survey compared to 37% in the Transvaal. However, cataract still remains an important cause of vision loss in this setting and continued efforts are required if the target of eliminating avoidable blindness by 2020 is to be achieved. Encouragingly cataract surgical outcomes met the WHO recommend target of a good outcome (VA>6/18) for ≥80% of eyes with uncorrected vision. Refractive error was the leading cause of visual impairment which suggests the need for increased optical services in this setting.

There was clear variation in the prevalence of vision loss between different socio-economic groups, with the highest prevalence among people with lowest household SES and those lacking in formal education. This could be due to a higher incidence of blinding conditions in these groups. However, there was also evidence of disparity in utilisation of ophthalmic services which may explain this variation: among people needing cataract surgery at VA<6/60, only 67% of the poorest tertile had received surgery compared to 100% of the wealthiest tertile. Our findings support those in Pakistan [21], India [20] and America [33] which show poverty to be a predictor of a higher prevalence of blindness and poorer access to ophthalmic services. Vision loss may also contribute to poverty – a multicentre study showed that after sight restoring cataract surgery, people were more likely to engage in productive activities and household expenditure increased compared to before surgery [18], [19]. Lack of formal education remained a significant predictor of vision loss even after multivariate adjustment suggesting some effect of this variable independent of SES. The association between vision loss and lack of education has been noted elsewhere [34]. Our findings concur with previous studies in South Africa which have shown disparities in health status [35], [36], [37] and access to health services [22], [23], [38] among different socio-economic groups.

Interestingly, just over a third of people in the poorest tertile reported attending private health facilities for their cataract surgery. The reasons for this are unclear but may be due in part to high participation in medical insurance schemes (which all cover cataract surgery) among people in any kind of formal employment and the fact that in the public services cataract surgery is only undertaken for people with VA<6/18 in the better eye.

The trend of higher risk of vision loss among women observed in this study has been noted elsewhere [39]. This may be due to longer life expectancy among women as many eye diseases are age related, increased susceptibility to certain ophthalmic conditions or gender disparities in access to and utilisation of health services. In our study the association was no longer significant after adjustment for other socio-economic factors.

There were limitations in this study. Due to the simplified examination protocols used in RAAB it is not always possible to classify posterior segment diseases into different aetiologies. We therefore grouped posterior segment diseases together for discussion purposes. The refusal rate (5.5%) was significantly higher in Cape Town compared to other SSA studies [4], [5], [7], [9]. This may reflect the middle income profile of Cape Town with participants placing less value on a “free eye test”. Due to safety concerns, we had to reselect two clusters. We cannot rule this out as a source of bias, although the replacement clusters were from neighbouring areas with similar socio-economic characteristics. We used cluster sampling because simple random sampling is not feasible in such large populations given time and resource constraints and the lack of lists of all individuals in the population. Cluster sampling may introduce a higher sampling error, however our sample size calculation was inflated to adjust for the expected design effect from this sampling approach. Finally, this survey may underestimate the actual demand for cataract services in the public sector because patients from the surrounding districts and provinces commonly travel to Cape Town for cataract surgery.

There were also strengths. Additional efforts were made to revisit clusters at the weekend to in order to examine people who were working or away during the week days. The resulting response rate was high which reduces selection bias. We used standardised approaches to collect ophthalmic and socio-economic data. To the best of our knowledge this is the first survey in the region to examine the relationship between vision loss and socio-economic factors.

### Conclusions

The prevalence of blindness in people ≥50 years in Cape Town was lower than expected probably because of high cataract surgery coverage. The contribution of posterior segment diseases as a cause of blindness is higher than previously reported in South Africa and SSA. There were socio economic disparities in prevalence of vision loss and cataract surgical coverage. These inequalities need to be addressed in order for Vision 2020 targets to be achieved in Cape Town.
